# An assessment of implementation science research capacity in Uganda

**DOI:** 10.1186/s12961-020-00653-2

**Published:** 2021-02-08

**Authors:** Aggrey S. Semeere, Fred C. Semitala, Olivia Lunkuse, Anne Katahoire, Nelson K. Sewankambo, Moses R. Kamya

**Affiliations:** 1 Infectious Diseases Institute, Makerere University, P.O. Box 22418, Kampala, Uganda; 2 Implementation Science programme, Department of Medicine, Makerere University, Kampala, Uganda; 3 Makerere University Joint AIDS programme, Kampala, Uganda; 4 Child Health and Development Centre, Makerere University, Kampala, Uganda; 5 School of Medicine, Makerere University College of Health Sciences, Kampala, Uganda

**Keywords:** Implementation science research training, Sub-Saharan Africa, Resource-poor settings, Curriculum development, Uganda

## Abstract

**Background:**

In Uganda and other resource-poor countries, relevant research findings face a tortuous path to translation into policy and routine practice. Implementation science (ImSc) research could facilitate faster translation. Presently it is unclear what ImSc research capacity and possible training needs exist among Ugandan researchers. To assess both components, we interviewed potential trainees in Kampala, Uganda.

**Methods:**

We used a cross-sectional design to survey potential ImSc trainees who had some research training and involvement in generating or utilizing research. Using a questionnaire, we documented eligibility for ImSc training, knowledge and interest in training, existing self-assessed confidence in initiating clinical research (SCICR) and self-assessed confidence in initiating ImSc research (SCIIR), availability for training and preferred modes of training. We developed scores from the Likert scales and used descriptive statistics, logistic regression and ordinal logistic regression to evaluate predictors of SCIIR.

**Results:**

Between November 2016 and April 2017, we interviewed 190 participants; 60% were men, with a median age of 37 years. Among participants, 33% comprised faculty, 37% were graduate students and 30% were project staff. The majority of respondents knew about ImSc (73%) and were research-trained (80%). Only 9% reported any ImSc-related training. Previous ImSc training was associated with higher odds of a SCIIR score ≥ 75th percentile. Previous ImSc training compared to not having any training was associated with higher odds of reporting abilities in behaviour change theory integration (OR: 3.3, 95% CI: 1.3–8.5, *p* = 0.01) and framework use in intervention design and implementation (OR: 2.9, 95% CI: 1.1–7.4, *p* = 0.03), accounting for age, sex and current employment. In addition, 53% of participants preferred in-person (face-to-face) short ImSc courses compared to a year-long training, while 33% preferred online courses. Participants reported median availability of 6 hours per week (IQR: 4, 10) for training.

**Conclusion:**

Most participants had some understanding of ImSc research, had research training and were interested in ImSc training. Those with previous ImSc training had better skills and SCIIR, compared to those without previous training. A hybrid approach with modular face-to-face training and online sessions would suit the preferences of most potential trainees.

## Introduction

Despite generating significant health research findings, many resource-poor countries, like Uganda, have dismal health indices in several areas. Any attempt to compare health indices of Uganda against proven research interventions quickly reveals tremendous gaps between what is known to optimize health and healthcare, and what actually happens in practice [1–3]. If properly implemented, many proven research findings could address critical local and regional health challenges, and radically transform population health. For instance, in a number of resource-poor countries, current practice has yet to match research evidence to prevent new HIV infections with efficacious prevention strategies [4–10] and achieve optimal HIV treatment targets [11, 12]. Creating evidence-based disease-focused guidelines is a common approach to enhance uptake, but on their own, guidelines cannot lead to effective implementation. Similar evidence–practice (implementation) gaps exist for malaria control [11]; tuberculosis (TB) case finding [12], diagnosis and infection prevention [13, 14]; and noncommunicable disease (NCD) prevention, diagnosis and treatment [15].

Tackling Africa and Uganda’s health care challenges could require a significant investment in implementation science (ImSc) research to bridge the gap between evidence and practice. Elsewhere researchers and practitioners have embraced ImSc research, with its systematic focus on enhancing uptake of evidence-based interventions to improve population health [16, 17] while discouraging unbeneficial health practices [18]. Multidisciplinary teams are required to carry out ImSc research utilizing specialized skills to clarify the implementation context, engage stakeholders, design theory- and stakeholder-informed interventions, and perform rigorous theory-based evaluations in real-world settings. Therefore, establishing ImSc research capacity requires training to obtain these various unique skills. Such training is available primarily in resource-rich settings [19–22], and is scarce in resource-poor countries [23, 24]. Training programmes in North America and Europe are largely inaccessible to most trainees from resource-poor countries, mainly due to high costs. The handful of training programmes that exist in sub-Saharan Africa are also inaccessible to many potential trainees because of the need to travel to other countries for training [24]. Online training opportunities exist, but these usually offer didactic material and no mentorship to enable one to initiate and satisfactorily implement an ImSc  research project [25]. Therefore, to inform the relevance of ImSc research training in Uganda and gauge its potential uptake, we surveyed potential academic and nonacademic trainees to understand current ImSc research capacity and training needs. The findings from this survey provided guidance for the development of an ImSc training programme at the Makerere University College of Health Sciences (MakCHS).

## Methods

### Overall design

Between November 2016 and April 2017, we performed a cross-sectional survey of potential ImSc trainees and faculty to describe the following: self-assessed confidence in initiating clinical research and ImSc research; ImSc knowledge, previous training and interest in training; availability to participate in training; and preferred modes of training. We also evaluated the association between previous ImSc training and self-assessed confidence in various ImSc research skills.

### Study site, participants and sampling

Survey participants included graduate students, lecturers (university faculty) and non-academicians (associated with clinical programmes or research projects and health administrators). Participants were drawn from various institutions in Kampala that included Makerere University, the Uganda Ministry of Health, HIV care and treatment projects, and research institutions. At Makerere University, graduate students and faculty were from the School of Medicine, School of Health Sciences, School of Public Health and College of Humanities and Social Science. programme/project staff were from Makerere University Joint AIDS programme (MJAP), Makerere University–Johns Hopkins University (MUJHU) collaboration, the Infectious Diseases Institute (IDI), The AIDS Support Organization (TASO), Centers for Disease Control and Prevention (CDC)-Uganda and the Ministry of Health.

Eligibility was premised on having participated in or obtained prior foundational clinical research training [20]. We targeted eligible health personnel including: 1) graduate students, 2) faculty/lecturers and 3) employees with a master’s degree or equivalent in a clinical, administrative or research programme (project/programme staff). Working with the respective human resource offices (faculty or employees) and registrars (graduate students) at the above-mentioned institutions, we first identified eligible individuals at the institutions using available records and found 1340 in total. For each institution we then determined what proportion of the intended sample of the 207 participants to draw depending on the size of the institution and contribution to the total. From the lists of those eligible, we then randomly selected participants using Mircosoft Excel, and the process was repeated until we obtained the target sample size to contribute the institution’s proportion. Identified participants were contacted and requested to provide informed verbal consent for a 20- to 30-minute face-to-face interview conducted by a trained research associate. Identified individuals whom we failed to reach or who declined to participate were replaced by another randomly selected eligible individual.

The survey protocol was approved by the Makerere University School of Medicine Research and Ethics Committee and by the Uganda National Council for Science and Technology (Registration number HS2130).

### Measurements

All consenting participants were interviewed and responses recorded on a questionnaire. The questionnaire had sections that collected demographic information, previous clinical research training and practice information, self-assessed confidence in initiating clinical research (SCICR) scale and outputs, ImSc knowledge and interest in training, and self-assessed confidence in initiating ImSc research scale (SCIIR). The questionnaire was pretested on 12 potential trainees to access clarity, and a few corrections were made to the tool. Availability and preferred modes of training were evaluated only among those interested in training. Specific details are outlined below (Additional file 1).

SCICR We asked each participant to rate their confidence on a scale from 1 (very poor) to 5 (excellent) in various aspects (14 in all) required for clinical research project development and implementation, including the following: literature review, critiquing scientific evidence, generating hypotheses, choosing study designs, evaluating problems with study designs, methods to recruit and retain study participants, data collection methods, fundamentals of sample size calculation, choosing appropriate statistical analysis, principles of qualitative analysis, deriving conclusions from study results, presentation and delivery of scientific information, research leadership, and ethical conduct of research. The highest possible score from these 14 items was 70. Participants also provided information regarding research productivity that included the number of previous and ongoing studies, and publications, abstracts and grant funding.

*Knowledge of and interest in ImSc training.* Participants were asked to provide their understanding of what ImSc was. The answer was recorded as a structured response based on the ImSc definition. We defined ImSc research as “…the scientific study of methods to promote the systematic uptake of research findings and other evidence-based practices into routine practice, and, hence, to improve the quality and effectiveness of health services”, as suggested by Eccles et al. [17]. We also asked participants to inform us about any previous ImSc training and interest in ImSc research training.

*SCIIR* Participants graded their confidence in initiating ImSc research on a scale of 1 (very poor) to 5 (excellent), on six constructs that included the ability to set up multidisciplinary teams, identification of factors that influence implementation, community and stakeholder engagement, application of behaviour change theory to enhance or understand intervention uptake, using frameworks in the design and evaluation of interventions, and evaluation of effects after knowledge translation. The highest possible score from these six items was 30. These areas were selected based on core training constructs suggested by Gonzalez et al. [18].

*Availability and preferred modes of training.* Participants’ availability for training and how they preferred the training to be delivered, i.e. online, in-person or short course, were assessed only among those interested in ImSc training.

### Analysis

We generated descriptive summaries of participants’ charateristics and main findings as appropriate. We also produced summaries of ImSc definitions using descriptive statistics. SCICR and SCIIR scores were generated from each participant’s Likert scale item measurements. Initially for each of the SCICR and SCIIR scores, we first checked for reliability using Cronbach’s alpha, assessing whether the scales measured the same underlying concept (if *α* coefficient > 0.7). We then checked for inter-item correlation assessing whether individual questions in each scale resulted in consistent and appropriate responses (ideal range is 0.15–0.5). Lastly, we performed a factor analysis assessing whether scale items were loading on the same factor, i.e. all in one direction (eigenvalue > 1.2). We then generated each participant’s total score by obtaining the sum of the item scores for each scale. The study team had set the 75th percentile as the optimal threshold for both scores. We then estimated the 75th percentile using the total scores. Each participant’s total score for either scale was then dichotomized based on the 75th percentile. We dichotomized unique SCIIR item scores using the 75th percentile. We report the proportion of participants with an overall score ≥ 75th percentile for both SCICR and SCIIR scales. We also assessed the relationship between previous exposure to ImSc training (exposure) and having an overall SCIIR score ≥ 75th percentile (outcome) using logistic regression. We also further analysed the relationship between previous exposure to ImSc training (exposure) and the unique SCIIR item scores categorized as ≥ 75th percentile (outcomes) using ordinal logistic regression. Unadjusted and adjusted comparisons of the unique SCIIR items (outcomes) and odds of previous ImSc training (exposure) are reported. Adjusted models accounted for age, sex and stakeholder group. Analyses were performed using Stata version 13.1 and 16.0 software (Stata Corp., College Station, TX).

## Results

### Description of the surveyed population

Out of 1340 eligible participants, we sampled 207 individuals and eventually enrolled 190 (92%). We failed to enroll 17 participants, because 6 were not interested in the study, while 11 said they had insufficient time for the interview. Surveyed participants had a median age of 37 years (interquartile range [IQR]: 31, 44), and 115 (61%) were men. Sixty-three (33%) were university faculty, 70 (37%) graduate (masters and PhD) students, and 57 (30%) project staff (Table 1). More than three-quarters of the faculty were from the College of Health Sciences at Makerere University (76%), while most graduate students (71%) were pursuing master’s degree programmes; the remainder were enrolled in PhD programmes. Overall, 114 (60%) of those interviewed had a master’s degree as the most recent qualification, with 137 (72%) having attained their recent qualification after 2010 (Table 1).

**Table 1:** Characteristics of the participants interviewed to inform the development of the implementation science research training programme in, Kampala Uganda

Characteristic	Overall (*n* = 190)	University faculty (*n* = 63)	Graduate students (*n* = 70)	Project staff (*n* = 57)
Age, years	37 (31, 44)^a^	45 (41, 49)	31 (29, 33)	40 (36, 43)
Male sex	61%	71%	49%	63%
Recent qualification^b^				
Masters	54%	37%	–	74%
PhD	35%	60%	–	7%
Other	11%	3.2%	–	19%
Previous epidemiology training	81%	78%	76%	91%
Epidemiology qualification^c^				
Certificate	10%	10%	3.8%	17%
Postgraduate diploma	1.3%	–		0%
Master’s degree	11%	6.1%	0%	27%
PhD	3.3%	10%	0%	0%
None (just part of other training)	74%	74%	92%	56%
Epidemiology training duration, months	3 (2, 12)	3 (2, 12)	3 (2, 5)	4 (3, 24)
Any biostatistics training	80%	75%	71%	98%
Studies completed in last 3 years	2 (1, 4)	3 (2, 5)	1 (0, 3)	2 (1, 4)
Role in completed studies				
Study coordinator	10%	6.7%	2.5%	21%
Investigator	57%	75%	27%	59%
Research assistant	29%	1.7%	68%	7.1%
Biostatistician	3.4%	3.3%	0%	7.1%
Other^d^	0.6%	87%	2.5%	5.8%
No. of current research studies	1 (1, 3)	2 (1, 3)	1 (0, 1)	2 (1, 4)
Role in current research studies				
Study coordinator	6.8%	3.4%	0%	19%
Investigator	78%	83%	93%	58%
Research assistant	6.8%	3.1%	7%	2.4%
Biostatistician	2.7%	0%	0%	8.5%
Other^d^	5.7%	11%	0%	12%
At least one peer-reviewed publication	67%	98%	23%	88%
At least one abstract prepared	61%	84%	37%	65%
Grant applications ever attempted	53%	79%	19%	65%

^a^Median (interquartile range) unless indicated

^b^This applies only to university faculty and project staff

^c^For only the 154 with epidemiology training

^d^Other roles included data manager, medical officer, specialist/consultant, project leader, field coordinator

### SCICR scores and research outputs

**Fig. 1:**
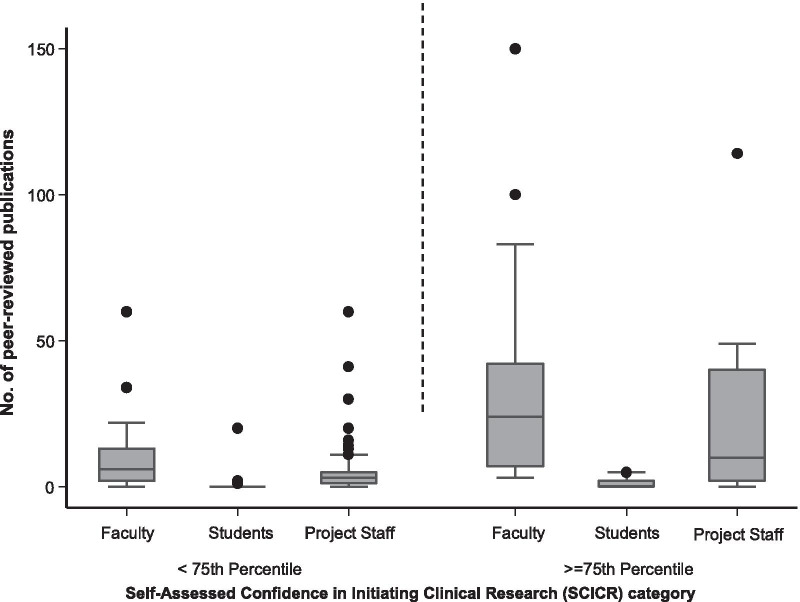
Box plot comparing of number of publications by category of self-assessed confidence in initiating clinical research (SCICR) score between various categories of participants surveyed

One hundred and fifty-four (81%) participants reported prior epidemiology training, while 152 (80%) reported biostatistics training (Table 1). The SCICR score scale was reliable, demonstrating Cronbach’s alpha of 0.93 and inter-item covariance of 0.36, with all items positively correlated with each other. Factor analysis also produced an eigenvalue > 1.2, suggesting that all scale items are loading on the same factor. Overall, participants had a median SCICR score of 53 points (IQR 46, 58) out of a possible 70 points (Table 2). Faculty had the highest median score of  58 (IQR 52, 64), while graduate students had the lowest  score of  48 (IQR: 42, 53). Only about a third of all the participants had a SCICR score ≥ 75th percentile. Compared to graduate students (13%) and project staff (19%), faculty had the highest proportion (52%), with a SCICR score ≥ 75th percentile (Fig. 1).

**Table 2:** Self-assessed confidence in initiating clinical research (SCICR) and self-assessed confidence in initiating implementation science research (SCIIR) scores among surveyed participants in Kampala, Uganda

Characteristic	Overall (*n* = 190)	University faculty (*n* = 63)	Graduate students (*n* = 70)	Project staff (*n* = 57)
*SCICR score*^a^				
Overall score	53 (46, 58)^b^	58 (52, 64)	48 (42, 53)	54 (48, 56)
≥ 75th percentile	28%	53%	13%	19%
SCIIR score^a^				
Overall score	21 (18, 24)	21 (19, 25)	21 (17, 24)	20 (18, 23)
≥ 75th percentile	28%	37%	27%	20%
*Item-specific SCIIR category ≥ 75th percentile*				
Multidisciplinary team formation	61%	75%	55%	54%
Factor identification to influence implementation	54%	64%	55%	41%
Community and stakeholder engagement	29%	34%	30%	77%
behaviour change theory integration	35%	37%	37%	29%
Framework use in intervention design and implementation	30%	44%	27%	14%
Evaluation of translation effects	42%	48%	44%	34%

^a^These scores were derived from Likert scales developed to assess participants’ research capabilities

^b^Median (interquartile range) unless indicated

The majority (81%) of participants had conducted research, with a median of three studies (IQR: 2, 5) within 3 years of the interview (Table 1). A similar proportion (78%) reported current involvement in research, with a median of two ongoing studies (IQR: 1, 3). Those who participated were mostly co-investigators (78%). In terms of research output, 68% of participants had at least one publication, while 61% had presented at least one abstract. Overall, the median number of publications was two (IQR: 0, 10), with faculty having the highest number of publications (10; IQR: 4, 30). Students had published the least, with 23% having at least one publication, compared to 98% of faculty and 88% of project staff (Table 1). Similarly, only 37% of postgraduates had ever prepared and presented an abstract, compared to 84% of faculty and 65% of project staff. Only about half (53%) of the participants had ever participated in a grant application, with the median number of applications being one (IQR: 0, 3) and participation being highest among faculty (79%).

### ImSc research capacity, previous training and SCIIR scores

Out of the 190 participants, 17 (9%) had participated in previous ImSc-related training. Training was via online courses, in-person short courses (1 day to 5 weeks), workshops, symposia, postgraduate course work, involvement in ImSc research projects and self-directed learning. The SCIIR was reliable given its Cronbach’s alpha of 0.89 and inter-item covariance of 0.52, with all items positively correlated with each other. Factor analysis also revealed an eigenvalue > 1.2, suggesting that all scale items are loading on the same factor. Overall, the median SCIIR score was 21 (IQR: 18, 24) out of a possible 30 points (Table 2) and did not differ by participant group. About a third of the participants (27%) had an overall SCIIR score ≥ 75th percentile. More faculty had a SCIIR score ≥ 75th percentile (35%) compared to graduate students (26%) and project staff (19%). Considering unique SCIIR items, community and stakeholder engagement (29%), behaviour change theory integration (35%), framework use in intervention design and implementation (30%), and evaluation of translation effects (sustainability) (30%) had the lowest proportion of participants with scores ≥ 75th percentile (Table 2). Further, participants with previous ImSc training were more likely to have an overall SCIIR score ≥ 75th percentile compared to those without, though this observation was not statistically significant after adjusting for age, sex and current position (odds ratio [OR]: 1.3, 95% CI: 0.4–4.2, *p* = 0.7). We observed a trend towards having a higher score for participants with previous ImSc training for most SCIIR items (Table 3). Previous ImSc training was associated with three times the likelihood of reporting competence in behaviour change theory integration (OR: 3.3, 95%CI: 1.3–8.5, *p* = 0.01) and framework use in intervention design and implementation (OR: 2.9, 95 %CI: 1.1–7.4, *p* = 0.03) after accounting for age, sex, and participant group (Table 3).

**Table 3:** Unadjusted and adjusted odds of having a score ≥ 75th percentile for the self-assessed confidence in initiating implementation science research (SCIIR) score and association with having obtained implementation science training among surveyed participants in Kampala, Uganda

Score categories	Unadjusted		Adjusted*	
	Odds ratio (95% CI)	*P* value	Odds ratio (95% CI)	*P* value
Overall SCIIR score above 75th percentile	1.2 (0.4, 3.7)	0.8	1.3 (0.4, 4.2)	0.7
Multidisciplinary team formation score	0.9 (0.3, 2.1)	0.8	0.9 (0.4, 2.2)	0.8
Factor identification to influence implementation score	1.2 (0.5, 3.1)	0.6	1.4 (0.5, 3.2)	0.5
Community and stakeholder engagement score	1.7 (0.6, 4.2)	0.3	1.7 (0.7, 4.3)	0.2
behaviour change theory integration score	**3.0 (1.1, 7.5)**	**0.02**	**3.3 (1.3, 8.5)**	**0.01**
Framework use in intervention design and implementation score	**2.8 (1.1, 7.2)**	**0.04**	**2.9 (1.1, 7.4)**	**0.03**
Evaluation of translation effects score	1.1 (0.4, 2.7)	0.9	1.1 (0.5, 2.9)	0.8

Bold values are statistically significant* p* < 0.05

*Adjusted for primary position, sex and age at interview using logistic regression with the outcome being obtaining a score ≥ 75th percentile and the exposure as prior implementation science training

### Knowledge and interest in ImSc training

A majority of the participants, 139 (73%), were aware of ImSc research. Compared to 55 (87%) faculty and 52 (91%) project staff, only 31 (44%) graduate students had heard about ImSc research. The majority of participants (81%) were able to provide a definition of what they thought ImSc research encompassed, mentioning at least one out of the four major components of the definition, as summarized in Table 4 [17]. Ten percent either stated an insufficient ImSc definition or provided a definition that did not encompass any of the four major components. Nine percent stated that they did not know what ImSc research was. Almost all (182, 96%) participants expressed interest in ImSc training irrespective of stakeholder group.

**Table 4:** Themes defining implementation science given by potential implementation science research trainees in Kampala, Uganda

Themes used in defining implementation science	*N* = 139^a^
Involves translation of proven or evidence-based health innovations, policies	54%
Occurs in routine care or public health settings	37%
Uses scientific methodologies	23%
behaviour change or change is required in current practice	16%
Don’t know what implementation science is	10%

^a^This represents participants who expressed interest in implementation science training

### Preferred modes of training and availability

Among participants interested in ImSc training, 91 (53%) preferred ImSc training delivered as a series of face-to-face short courses over a year, while 56 (33%) preferred online courses over the same period. Some participants had a preference for blended learning, with online coursework running alongside in-person interaction (62%). Sixty-eight percent of faculty preferred in-person short courses, compared to 44% of graduate students and 48% of project staff. Further, 17 (34%) project staff preferred online training, compared to 11 (20%) faculty and 7 (10%) graduate students. Overall, a majority preferred a combination of online coursework with in-person interaction (69% project staff, 64% of graduate students and 50% faculty). Availability for ImSc training was reported as a median of 6 h per week (IQR: 4, 10). Project staff were most available (6.5 h per week [IQR: 4, 10]) for ImSc training.

## Discussion

We interviewed academic and nonacademic stakeholders, who are potential ImSc trainees, to clarify current ImSc research capacity and training needs. Our approach was motivated by the need to develop an informed and contextualized ImSc training programme, adapted to the needs of trainees and based on resources available. Very few participants had received formal ImSc training, and the expressed confidence in initiating ImSc research was generally low.

We studied a diverse and representative sample of potential trainees. The most recent documentation of capacity building for ImSc in East Africa assessed just a handful of highly selected participants, since they were already enrolled as fellows in a training programme [23]. Our results represent a larger pool of potential trainees who represent the range of the ImSc research stakeholder cadre in Uganda and sub-Saharan Africa. Our results show that over three-quarters of participants had previous research training. This was corroborated by research productivity and confidence in initiating clinical research, especially among faculty, who had the highest number of peer-reviewed publications compared to the other groups (Table 1). While graduate students had been exposed to research, only a few had evidence of research outputs (Table 1). Being a subspecialty research field, ImSc requires considerable knowledge and practice of research as a foundation from which to initiate ImSc research training [19, 20]. Generally, research competence is required by ImSc researchers to gauge the quality of research evidence prior to determining its readiness for translation. Also, ImSc research typically utilizes conventional research methodology for study design and analysis. Having sufficient research training and/or exposure is therefore a prerequisite. Our findings suggest that most faculty and project staff possessed what could be considered the prerequisite research competence to initiate ImSc research training, compared to only a small proportion of graduate students.

ImSc research is a team science that relies on multiple skills derived from many disciplines [18, 26]. We therefore assessed participants’ confidence in various ImSc disciplines (items of the SCIIR scale) (Table 2) as suggested by Gonzalez et al. [18]. Overall, about a tenth of interviewees reported previous exposure to ImSc training, and about a third had obtained ImSc skills (SCIIR score > 75th percentile) from non-ImSc training, since these skills are not exclusive to ImSc. Importantly, only participants with previous ImSc training had higher odds of having better scores for “behaviour change theory integration” and “framework use in intervention design and implementation”. So, while it is possible that the other components could be obtained elsewhere, behaviour change theory integration and framework use in intervention design and implementation are important to implementing and evaluating ImSc research, suggesting a need for training. Focused ImSc training, bringing together the diverse required skills, would enable the blending of individual skill sets to facilitate ImSc research implementation. Importantly, having had previous exposure to these concepts and approaches could simplify the assimilation process in the context of ImSc. Likewise, participants with existing skills in these disciplines could constitute a pool of trainers, facilitators or mentors for the ImSc-specific courses, especially in resource-poor settings where only a few are trained. The set-up of an ImSc training programme in a resource-poor setting should, therefore, consider the diverse ImSc skills available among local faculty as opposed to looking for fully ImSc-trained faculty.

We found that the concept of ImSc research was not new to most participants, since 81% of interviewees could provide an acceptable definition of ImSc research (Table 4). Awareness and interest in ImSc were, however, not matched by confidence among those who had some prior training, since participants felt they needed more training. Based on reported modes of training received (mostly short-term and online), participants seemed to be inadequately empowered to launch full-fledged ImSc research. This observation indicates a need for both didactic training and mentorship. It is worth noting that interest in ImSc research training is currently driven in part by research funding opportunities and programmes, which require applicants to roll out evidence-based interventions for better health outcomes [27]. Consequently, faculty and researchers who do not have the required skill set are the ones most likely to respond to the training opportunities to take advantage of these funding opportunities.

Participants also provided valuable responses to guide training delivery. In-person short courses, online courses and a hybrid approach (in-person with online courses) were preferred in that order. Those interested in training were willing to set aside time despite some having full-time jobs. Delivery of the training should therefore accommodate trainees’ time availability to ensure maximum benefits. A hybrid approach mentioned above seems to be a reasonable compromise, though the duration of the modules and in-person sessions will have to be re-evaluated against course content.

Our findings should be considered with some limitations in mind. We relied on subjective self-reported responses; hence our results could have been prone to social desirability bias. We attempted to minimize this by ensuring anonymity and confidentiality for each of the respondents. Our assessment of research capacity was also limited to previous exposure to quantitative research, and we did not inquire about qualitative research. Since there is generally limited expertise and less implementation of qualitative research in the region, our results in general reflect the regional research environment in which there is more of an emphasis on quantitative than on qualitative research. Also, our Likert scales were designed for this survey and hence may lack wider use and validation. Nonetheless, they were consistent, since they evaluated constructs as intended based on the reliability analysis. They might need further validation if they are to be used elsewhere. Regression analyses are also prone to residual confounding from unmeasured and unknown confounders.

## Conclusion and implications

The majority of participants we assessed had some understanding of ImSc research, were interested in ImSc training and had prerequisite research training. Prior exposure to ImSc-related skills, with no ImSc-specific training, was not sufficient to provide confidence to initiate ImSC research. We also identified ImSc disciplines where skills exist, hence a potential source of faculty. Conversely, we also noted the disciplines in which potential trainees were deficient in required ImSc skills. A hybrid training approach consisting of modular in-person courses with online support combined with mentorship would be critical in building ImSc research competence. Finally, we believe that our findings represent responses of potential trainees from a resource-poor African setting, and hence they can inform relevant ImSc training needs in the region.

## Supplementary information

**Additional file 1.** Questionnaire.

## Data Availability

The datasets used and/or analysed during the current study are available from the corresponding author upon request.

## Abbreviations

AIDS: Acquired immunodeficiency syndrome
ART: Antiretroviral therapy
CDC: Centers for Disease Control and Prevention
HIV: Human immunodeficiency virus
ImSc: Implementation science
IDI: Infectious Diseases Institute
IQR: Interquartile range
MJAP: Makerere University Joint AIDS programme
MUJHU: Makerere University–Johns Hopkins University collaboration
OR: Odds ratio
SCICR: Self-assessed confidence in initiating clinical research
SCIIR: Self-assessed confidence in initiating implementation science research
TASO: The AIDS Support Organization
TNA: Training needs assessment
