# Supplementary material for: An assessment of implementation science research capacity in Uganda
Source: Health Res Policy Syst. 2021 Feb 8;19:14. doi: 10.1186/s12961-020-00653-2 (PMC7869465; doi:10.1186/s12961-020-00653-2)
Supplement: Supplementary file 1 — Additional file 1. Questionnaire. [file 12961_2020_653_MOESM1_ESM.pdf]

Participant ID#	Date of Interview	Interviewer's Initials
☐☐☐☐☐☐☐	☐☐/☐☐☐/☐☐☐☐<br>DD MMM YYYY	☐☐☐

IMPLEMENTATION SCIENCE NEEDS ASSESSMENT QUESTIONNAIRE

Demographic Information

1. What is your date of birth	2. Sex	3. What is your current primary position at Makerere University?
☐☐/☐☐☐/☐☐☐☐<br>DD MMM YYYY	☐ Male<br>☐ Female	☐ Faculty<br>☐ Postgraduate→ *Skip to 5*<br>☐ Project staff<br>☐ Other Non Makerere staff
**4. If Faculty, Project staff or Other Non-Makerere staff, Are you also a postgraduate student?**	**5. What is your level of Postgraduate training?**	**6. What Makerere University department were you are attached to as a Postgraduate student?**<br>*Go to 11 if project or Non Makerere staff.*
☐ Yes<br>☐ No→ *Skip to 7*	☐ Master's Student<br>☐ PhD student	______________________________
**7. Within which College of Makerere University are you a faculty?**<br>*Skip if postgraduate student, project or Non Makerere staff*	**8. What School within Makerere University are you faculty?**<br>*Skip if postgraduate student, project or Non Makerere staff*	**9. What department do you belong to?**<br>*Skip if postgraduate student, project or Non Makerere staff*
______________________________<br>______________________________	___________________________<br>___________________________	______________________________<br>______________________________
**10. What is your Faculty position?**<br>→ *Skip to 13 for Faculty*	***For only Project staff***<br>**11. Where do you hold the primary position?**<br>*Skip for all Faculty*	***For only Project staff***<br>**12. What is your position at our place of work?**<br>*Skip for all Faculty*
☐ Assistant Lecturer<br>☐ Lecturer<br>☐ Senior Lecturer<br>☐ Associate Professor<br>☐ Professor	___________________________<br>___________________________	___________________________<br>___________________________
**13. What is your most recent Academic qualification?**	**14. Which year did you obtain this qualification?**	**15. Where did you obtain this qualification?**
☐ Master's degree<br>☐ PhD	☐☐☐☐	☐ Makerere University<br>☐ Other University in Uganda<br>☐ Other University in Africa<br>☐ Other University in Europe<br>☐ Other University in US<br>☐ Other University in Asia<br>☐ Other University in Australia

Participant ID#	Date of Interview	Interviewer's Initials
☐☐☐☐☐☐☐	☐☐/☐☐☐/☐☐☐☐<br>DD MMM YYYY	☐☐☐

Previous Clinical research training and current practice

**16. Have you had any training in epidemiology?**	**17. What was the duration of Epidemiology training?**	**18. What was the nature of the Epidemiology training?**
☐ Yes<br>☐ No ⟶ *Skip to 20*	☐☐☐ Days<br>☐☐☐ Weeks<br>☐☐☐ Months<br>☐☐☐ Years	☐ Specialized Master degree<br>☐ Specialized PhD degree<br>☐ Course undergraduate<br>☐ Course unit in Master's training<br>☐ Course unit in PhD training<br>☐ Certificate course
**19. What qualification did the Epidemiology training lead to?**	**20. Have you had any training in Biostatistics?**	**21. What was the nature of the Biostatistics training?**
☐ Certificate<br>☐ Master's Degree<br>☐ PhD<br>☐ None (just part of other training)	☐ Yes<br>☐ No ⟶ *Skip to 22*	☐ Specialized Master degree<br>☐ Specialized PhD degree<br>☐ Course undergraduate<br>☐ Course unit in Master's training<br>☐ Course unit in PhD training<br>☐ Certificate course
**22. How many concluded research studies have you participated in the past 3 years?** *If answer is 0, Go to 26*	**23. Of these studies, how many did you initiate on your own?** *Skip if answer is 0 in 22*	**24. How many were initiated by others and you just participated?** *Skip if answer is 0 in 22*
☐☐☐	☐☐☐	☐☐☐
**25. For the studies in 22 above, what was your role?** *Skip if answer is 0 in 22*	**26. How many ongoing studies are you currently involved in?** *If answer is 0, Go to 28*	**27. For the studies in 26 above, what was your role?** *Skip if answer is 0 in 26*
☐ Study coordinator<br>☐ Investigator<br>☐ Research assistant<br>☐ Biostatistician<br>☐ Other:______________________	☐☐☐	☐ Study coordinator<br>☐ Investigator<br>☐ Research assistant<br>☐ Biostatistician<br>☐ Other:______________________
**28. How many peer reviewed research publications have you participated in writing?**	**29. Of these, for how many are you the first author?** *Skip if answer is 0 in 28*	**30. How many abstracts have you prepared and presented in the last 2 years?**
☐☐☐	☐☐☐	☐☐☐
**31. How many grant applications have you prepared in the last 2 years?** *If answer is 0 ⟶ Skip to 34*	**32. Of these how many were funded?** *Skip if answer is 0 in 31*	**33. Of the applications in 31, how many did you initiate?** *Skip if answer is 0 in 31*
☐☐☐	☐☐☐	☐☐☐

34. Self-evaluation of research core competencies:
On a scale of 1-5, with (1 being very poor and 5 is excellent) how would you grade yourself in terms of research core competencies listed. For each competency choose one response.

Core competency	Excellent (5)	Good (4)	Fair (3)	Poor (2)	Very Poor (1)
a. Extracting literature from appropriate bibliographic sources.	☐	☐	☐	☐	☐
b. Critiquing clinical and scientific evidence derived from literature.	☐	☐	☐	☐	☐
c. Generating relevant biomedical, clinical, public health, or translational research hypothesis.	☐	☐	☐	☐	☐
d. Choosing the appropriated research Designs to address generated research questions	☐	☐	☐	☐	☐
e. Evaluating possible problems in the design and execution of a study	☐	☐	☐	☐	☐

Participant ID#	Date of Interview	Interviewer's Initials
☐☐☐☐☐☐☐	☐☐/☐☐☐/☐☐☐☐<br>DD MMM YYYY	☐☐☐

f. Developing appropriate methods to recruit and retain study participants for a selected research design	☐	☐	☐	☐	☐
g. Utilize appropriate data collection methods to generated data required both qualitative and quantitative research	☐	☐	☐	☐	☐
h. Applying fundamental principles of statistical analysis, such as power analysis, correlation, causation, regression, and summary statistics	☐	☐	☐	☐	☐
i. Apply appropriate principles for qualitative data analysis	☐	☐	☐	☐	☐
j. Selecting the appropriate statistical approach for the interpretation of preclinical and clinical datasets	☐	☐	☐	☐	☐
k. Developing appropriate conclusions based on results from research data	☐	☐	☐	☐	☐
l. Presentation and Delivery of Oral and Written Scientific Information	☐	☐	☐	☐	☐
m. Scientific Leadership, Management, and Cross-Disciplinary Teamwork in study design and implementation	☐	☐	☐	☐	☐
n. Ethical Conduct in the design and implementation of studies.	☐	☐	☐	☐	☐

Awareness, knowledge about and interest in ImSc training

35. Have you heard about Implementation Science?

☐ Yes
☐ No ⟶ *Skip to 45*

36. What have you heard about ImSc?

____
____
____

37. How did you get to hear about ImSc?

____
____
____

38. Can you define Implementation science?

____
____
____

39. Have you had any training in Implementation Science?

☐ Yes
☐ No ⟶ *Skip to 45*

40. Describe the training have you received?

____
____
____

41. Where did you receive this training?

____
____

42. Did you attain any qualification at the end of the training, if so what was it?

____
____

43. Describe what you learnt during your training in ImSc?

44. What areas of ImSc are you currently using in your research?

Participant ID#	Date of Interview	Interviewer's Initials
☐☐☐☐☐☐☐	☐☐/☐☐☐/☐☐☐☐<br>DD MMM YYYY	☐☐☐

45. With or without previous training, would you be interested ImSc training or skills?

☐ Yes ⟶ Reasons for interest?

☐ No ⟶ Reasons for no Interest?

End Questionnaire here if **Answered NO to 45**

46. What would the impact of this training be on your current work and career path?

47. If Yes, how important was this training to your career. On a scale of 1-3 (not so important-3 Very important) please grade importance of the ImSc training?

☐ Very important
☐ Somewhat important
☐ Not so important

48. **Existing level of core ImSc proficiencies among target trainees.**
For the following set of questions we are going to inquire about your proficiency in specific skills that are required in ImSc. On a Scale of 5-Excellent to 1-Poor, how well do you assess your skills in these areas?

Core ImSc competency	Excellent (5)	Good (4)	Fair (3)	Poor (2)	Very Poor (1)
a. Formation of multidisciplinary teams that blend relevant disciplines and skills to address and implementation problem	☐	☐	☐	☐	☐
b. Ability to identify a range of factors, behavioral, socio-ecological, institutional, etc... that inform implementation questions and problem prioritization?	☐	☐	☐	☐	☐
c. Ability and skills to build relationships with communities and stakeholders to engage in multiple approaches?	☐	☐	☐	☐	☐
d. Ability to Integrate behavior change theories into various intervention designs to enhance translation?	☐	☐	☐	☐	☐
e. Ability to select frameworks that reflect diverse views to guide intervention design and implementation	☐	☐	☐	☐	☐
f. Able to select appropriate outcome and impact measures to evaluate effects of translational activities?	☐	☐	☐	☐	☐
